# Development and evaluation of a web-based toolkit to inform mental health professionals about digital mental health interventions for eating disorders

**DOI:** 10.1186/s40337-025-01518-1

**Published:** 2026-01-09

**Authors:** Gwendolyn Mayer, Diana Lemmer, Benita Gräfin von Koenigsmarck, Hans-Christoph Friederich, Stephanie Bauer

**Affiliations:** 1 Department of General Internal Medicine and Psychosomatics, Heidelberg University Hospital, Heidelberg, Germany; 2 Center for Psychotherapy Research, Heidelberg University Hospital, Heidelberg, Germany; 3 Institute of Psychology, Ruprecht-Karls University of Heidelberg, Heidelberg, Germany; 4 German Center for Mental Health (DZPG), Partner site Mannheim-Heidelberg-Ulm, Germany

**Keywords:** Mental health care, Eating disorders, Mixed methods, Implementation, Toolkit, Mental health practitioners, Mental health professionals, Digital mental health

## Abstract

**Introduction:**

Eating disorders (EDs) are serious mental disorders that often remain untreated for many years. Digital mental health interventions could provide low-threshold support especially in underserved areas. However, the knowledge of mental health professionals (MHPs), who could integrate such interventions into routine care, is still limited.

**Objective:**

This study aimed at the development and evaluation of a web-based toolkit to provide evidence-based knowledge about digital interventions for EDs for MHPs.

**Methods:**

A mixed methods design was chosen to iteratively evaluate the toolkit. First, three focus groups with 16 MHPs were conducted, who tested the toolkit and gave feedback. Then, *N* = 66 MHPs completed an online survey with self-developed questionnaires on the perceived quality of the toolkit and its modules. Further, several scales were used to measure website clarity (Web-CLIC), visual aesthetics (VisAWI-S), system usability (SUS), e-therapy attitudes (ETAM) and elements of the Unified Theory of Acceptance and Use of Technology (UTAUT).

**Results:**

The focus groups resulted in a number of suggestions that were used to improve the toolkit. The results of the quantitative study showed that the toolkit reached high ratings in website clarity, visual aesthetics and system usability. E-therapy attitudes were positive in 51 (77.3%) cases. While perceived usefulness was M = 2.75 (SD = 0.62, range = 1-3.86), relative advantage remained lower with M = 1.08 (SD = 0.54, range = 0-2.5). Results following the concept of UTAUT, showed a medium Behavioural Intention to use digital interventions for their patients with M = 3.60 (SD = 1.01) and a medium Performance Expectancy of M = 3.50 (SD = 0.93) with lower values for anorexia nervosa than for other kinds of EDs.

**Conclusions:**

In general, MHPs show positive attitudes towards the use of digital interventions for patients with EDs to complement conventional psychotherapy. However, there is also a clear need for more knowledge in the field. The web-based toolkit may serve as a promising educational resource to meet the needs of MHPs. The effectiveness of the toolkit regarding learning success can be tested in future studies.

**Supplementary Information:**

The online version contains supplementary material available at 10.1186/s40337-025-01518-1.

## Abstract (plain language summary)

Eating disorders are serious mental disorders that often remain untreated for many years. Digital mental health interventions are therapeutic services for people with a mental disorder that can be delivered on electronic devices. They may support people in remote areas with limited access to a therapy. As these interventions are new, only few mental health professionals have sufficient knowledge about them. A web-based toolkit was developed to provide knowledge about digital interventions for eating disorders to them. The toolkit was evaluated through group discussions with mental health professionals. The toolkit was then improved on the basis of the results. In the next step, 66 mental health professionals filled in an online survey and answered questions referring to their opinions about the usefulness and relevance of the toolkit. The professionals showed generally positive attitudes towards the use of digital interventions for patients with eating disorders. However, there is also a clear need for more knowledge in this area. The web-based toolkit may serve as a promising educational resource to meet the needs of mental health professionals.

**Supplementary Information:**

The online version contains supplementary material available at 10.1186/s40337-025-01518-1.

## Introduction

### Background

Eating disorders (EDs) are serious mental disorders that are characterized by a high psychological and physical burden and a substantially increased mortality [1]. With a peak age of onset at 15.5 years in both, anorexia nervosa and bulimia nervosa and 19.5 years in binge eating disorder [2], they also have a huge impact on the quality of life for those affected and their families. In order to prevent chronicity, early treatment is crucial. However, only a minority of those affected seek appropriate help at an early stage and in many cases, several years pass between the onset of the illness and the start of treatment [3, 4]. The reasons for this treatment gap are, on the one hand, individual and attitudinal factors such as shame, stigmatization, lack of knowledge and the strong belief of not needing assistance [5]. On the other hand, there are structural and organizational barriers, such as a lack of specialized care close to home [6].

Searching for ways to enhance care for individuals with EDs, a large number of studies have been carried out over the past 20 years on the development and evaluation of digital interventions for EDs [7–9]. Benefits of this development have recently been expressed by mental health professionals (MHPs) in providing low-threshold access for vulnerable groups [10], however consensus was reached in preferring blended treatment concepts over stand-alone applications [11]. In Germany, the digital healthcare act (German: Digitale-Versorgung-Gesetz, DVG) was launched in 2019 and paved the way to warrant access to certified digital interventions (so-called DiGAs (Digitale Gesundheitsanwendungen)) for patients which are reimbursed by health insurance. However, recent reviews show that the evidence base concerning the effects of most DiGAs is still limited [12, 13]. Currently, only two of the available DiGAs target EDs [14].

Barriers to the uptake of digital interventions for mental health in general have been identified in primary care practitioners, who play a central role in prescribing digital health applications. A survey with 224 decision makers found limited intention or motivation to utilize them as core reasons for the lack of implementation [15]. More specifically, general practitioners and psychotherapists in Germany expressed to be not well informed about the development of digital mental health interventions [16]. Only few studies so far focus on clinician’s views on the integration of digital mental health in routine care for EDs, however, MHPs report only little experiences with these applications and see a high need to improve digital literacy in this field [17, 18].

To fill this gap and provide an easily accessible information source for MHPs on current developments and evidence on digital applications specific for EDs, a web-based toolkit was developed (available under: https://www.sida-essstoerungen.de/toolkit/). Similar toolkits in health care have been developed as an important strategy for quality improvements in different sectors [19]. Advantages include the wide reach, cost efficiency and the possibility to update the tool regularly. Last but not least, targeted information regarding specific diagnoses of EDs can be covered, as previous researchers have argued that certain patients suffering from chronic and severe anorexia benefit less from a digital treatment [25].

Besides from lacking knowledge, other factors can have an impact on the acceptance and uptake of digital health applications for EDs. Following the *Unified Theory of Acceptance and Use of Technology (UTAUT)* four key concepts explain why individuals (a) intend to use and (b) actually use a technology: *Performance Expectancy*,* Effort Expectancy*,* Social Influence*, and *Facilitating Conditions* [20].Regarding the acceptance of digital mental health in general, Performance Expectancy appears as a strong predictor across patient populations and MHPs [21]. More specifically related to ED-treatment, Anderson et al. showed high predictive values, again, for Performance Expectancy for the acceptance of digital dialectical behavioural therapy [22]. However, to the best of our knowledge, no study so far performed a systematic attempt to either inform MHPs on digital interventions for EDs and evaluate the willingness to integrate such interventions into the routine care of patients with EDs.

### Aims of the study

Based on recent findings, the present study aimed at the development and evaluation of a web-based toolkit to provide evidence-based knowledge about digital interventions for EDs and promote MHPs’ willingness to implement them, which is an essential prerequisite for the successful integration of such interventions into routine care. Another objective was to investigate differences between the intention to use digital interventions depending on the diagnosis of three most prevalent diagnoses anorexia nervosa, bulimia nervosa and binge eating disorder.

## Methods

### Study type

This study used a mixed methods research design to conduct an iterative evaluation of the toolkit for MHPs. The first, qualitative part of this study included focus groups with licensed MHPs and MHPs in training who tested the toolkit and gave direct feedback. The second part was an online survey with quantitative scales and open-ended questions. Both parts aimed at collecting feedback considering different dimensions of quality assessment.

Ethical approval was obtained by the Ethics Commission of the Medical Faculty at University of Heidelberg (S-082/2023).

### Recruitment

Recruitment followed a convenience sampling approach in both parts of the study. Personal contact networks, mailing lists, and social media channels were used to invite MHPs. Inclusion criteria were age above 18 years, sufficient German skills and being qualified to conduct mental health treatment either as a medical doctor, psychological psychotherapist or as a therapist in training. In order to participate in the study, it was required to have already treated patients, even for those participants who were still in training. Specific expertise related to the treatment of EDs, however, was not required.

### Characteristics of the toolkit

The toolkit of the current study is a multimedia online resource with informational texts, graphics, and videos. It is intended to inform current and prospective MHPs about the evidence base, areas of application as well as the opportunities and limitations of different digital applications for EDs. The materials use practical examples and experience reports to illustrate ways in which digital interventions may and may not be integrated into conventional treatment and which aspects need to be particularly considered from a therapeutic perspective.

The toolkit consists of six subsections focussing on EDs in general, areas of application for digital interventions, tips, digital health applications (German DiGAs), a glossary and literature. All sections began with highlights summarizing the key facts of the respective topic. The detailed descriptions in the various sections included videos with case examples and quotes from MHPs talking about their professional practice. The section describing EDs in general included a chapter with “9 truths about EDs” that we quoted from [23] and [24]. The intended use of the toolkit is broad and aims at MHPs with various levels of experience in the treatment of EDs. A more detailed description of the elements of the toolkits and its specific format with example illustrations is presented in supplementary material 1.

The development of the toolkit was carried out from February to July 2023 by a group of psychological and medical experts in the field of psychotherapy research and psychosomatics from the Heidelberg University Hospital. In order to ensure the quality of the materials presented in the toolkit, continuous feedback rounds were part of the development process.

### Part I: focus groups

A total of 16 MHPs were invited to focus groups. They were on average 32.07 years old (SD = 6.43) with 12 female (75.0%) and 4 male (25.0%) participants. The participants were allocated to three focus groups with five to six participants per group that were conducted online in July and August 2023 and took around 90 min each. After a general introduction to the aims of the study, the participants were given the opportunity to test the first version of the toolkit individually within 7 min. Then, feedback was collected regarding the format, design and content of the toolkit and discussed within the respective groups. The participants received an expense allowance for their participation (gift cards worth 100€). The feedback from the MHPs was prioritized in terms of the feasibility and objectives of the toolkit. Due to a lack of resources, it was not possible to implement all of the feedback on the videos, for example. However, almost all of the feedback on the content of the text was considered. Requests for recommendations for specific apps were ignored, as the toolkit was not designed for advertising purposes. Based on this process, the toolkit was adapted.

### Part II: online survey

In the second part of the study, an online survey was disseminated among MHPs between November 2023 and January 2024.

The survey started with the request to take 15–20 min for testing the toolkit prior to answering the questionnaires. Subsequently, a battery of different questionnaires was presented. One item asking whether participants had familiarized themselves with the toolkit prior to their participation was used to ensure their engagement. The completion of the questionnaires and testing of the toolkit required 30–40 min in total. An expense allowance was offered after completion of the survey in the form of a 50€ gift card.

A total of 74 people started to fill in the questionnaire, 70 participants completed it. Four participants neither had a treatment license of were therapists in training. Finally, 66 questionnaires were considered valid and were further analysed. The participants were aged M = 31.88 years (SD = 6.81) with 58 (87.9%) female and 8 (12.1%) male participants. Out of these participants, 60 (90.9%) had a background in psychology and 6 (9.1%) in medicine. The majority was in charge of treating adults (75.8%), while a minor group treated children and adolescents. For further demographic details see Table 1.

**Table 1:** Demographic characteristics of the survey participants

Characteristic	Category	Frequency (Percentage) *n* (%) Survey
Gender		
	Male	8 (12.1%)
	Female	58 (87.9%)
Professional background		
	Psychology	60 (91.9%)
	Medicine	6 (9.1%)
Status		
	Licensed	16 (24.2%)
	Still in professional training	50 (75.8%)
Clinical orientation		
	Cognitive behavioural therapy	42 (63.6%)
	Psychodynamic therapy	18 (27.3%)
	Systemic therapy	6 (9.1%)
Patient group*		
	Adults	50 (75.8%)
	Children & adolescents	16 (24.2%)
**Total**		**66 (100)**

Note. *The participants were asked for a specific qualification to treat children and adolescents. However, some of them might be in charge of treating both patient groups

### Measures

The online survey consisted of the following sections:

#### Demographics and technology skills

Demographic items asked for age, gender, and details on the professional background of the participants such as type of institution (hospital, outpatient services, residency, mixed), clinical orientation (cognitive behavioural therapy, psychodynamic therapy, systemic therapy) and specific training for certain age groups (adults or children and adolescents). In the next section, the participants were asked about their technology skills. Two questions referred to the age of first use of a computer and the internet. Moreover, the mean time spent per day on the internet and social media in private and professional life was asked. Finally, the degree of experience with digital media and devices was assessed by using module IC014 from the Information and Communication Technology (ICT) familiarity scale of the PISA study (Programme for International Student Assessment), a worldwide study carried out by the Organisation for Economic Co-operation and Development (OECD) [25]. These 5 items were scored from 1 to 4 with 1 indicating “strongly disagree” and 4 indicating “strongly agree”.

#### Evaluation of the toolkit

At first, a general evaluation was requested with six items developed for the purpose of this study. Three of them included general assessments with a Likert scale from 1 to 5 with 1 representing the lowest and 5 the highest degree of agreement to certain statements. The other three items were optional open-ended questions, where participants could indicate which content they missed, appreciated or disliked. Then, the specific modules of the toolkit were evaluated separately with four to six items depending on the number of features provided in the modules (e.g. videos, texts). The questions referred to clarity and usefulness of the content. Finally, specific components such as videos, textboxes, and images were assessed. The format of the items was the same as in the general section.

*Web-CLIC (Website - Clarity*,* Likeability*,* Informativeness*,* and Credibility)*: This questionnaire is a 12-item measure that asks for four main facets of users’ content experience: *clarity* (Cronbach’s α = .76), *likeability* (Cronbach’s α = .86), *informativeness* (Cronbach’s α = .88), and *credibility* (Cronbach’s α = .92). Items are assessed on a 7-point Likert scale from 1 (”do not agree at all”) to 7 (”fully agree”).

#### Short visual aesthetics of websites inventory (VisAWI-S)

This brief assessment instrument measures perceived visual aesthetics of websites. It consists of 4 statements where participants state their level of agreement on a 7-point Likert scale (ranging from 1 “strongly disagree” to 7 “strongly agree”; Cronbach’s α = 0.91) [26]. The authors of the instrument defined a threshold value of 4.5 indicating a rather positive perception of a website’s visual aesthetics [27].

#### System usability scale (SUS)

This standardized questionnaire has been widely used for the assessment of perceived usability. It consists of 10 items with a 5-point Likert scale (Cronbach’s α = 0.85) [28]. Analysing the SUS, a sum score ranging from 0 to 100 was calculated. A recent review on the use and interpretation of the SUS suggests a mean of 68 to be an average system usability, 74-78.8 to be B Grade and 78.9 to 100 A grade usability [29].

#### E-Therapy attitudes scale (ETAM)

The next section referred to more general attitudes towards digital interventions for mental disorders, starting with in an adapted version of the ETAM [30] which was designed to measure the subscales *Perceived usefulness and helpfulness* and *Relative advantage and comparability*. The instrument uses 17 items on a Likert scale from 0 (“strongly disagree”) to 4 (“strongly agree”). Cronbach’s alpha of the total questionnaire was α = 0.85. The two subscales Perceived usefulness and helpfulness and Relative advantage and comparability reached α = 0.78 and α = 0.76 respectively. In line with previous work [31], mean scores < 1.5 (a median score of 0 or 1) were defined as negative, values between 1.5 and 2.49 (median score of 2) as neutral, and scores ≥ 2.5 (median scores of 3 or 4) as positive attitudes toward guided internet interventions.

#### Unified theory of acceptance and use of Technology – UTAUT

The concepts B*ehavioural Intention*,* Performance Expectancy*,* Effort Expectancy*,* Social Influence*, and *Facilitating Conditions* were measured using 16 items developed by Philippi et al. [21] which were adapted for the purpose of this study. In short, Performance Expectancy refers to the belief that the use of the technology will lead to gains in job performance, Effort Expectancy means the degree of ease associated with the use of the technology, and Social Influence is defined as the belief that important others think that a person should use the technology. Finally, Facilitating Conditions include beliefs that organizational and technical infrastructure exists to support the usage of the technology [32]. All items used a 5-point Likert scale ranging from 1 (“does not apply at all”) to 5 (“applies completely”). The concepts Behavioural Intention and Performance Expectancy were asked separately for all three diseases anorexia nervosa, bulimia nervosa, and binge eating disorder. For the subscales Behavioural Intention Cronbach’s alpha amounted to α = 0.96, for Performance Expectancy to α = 0.97, for Effort Expectancy to α = 0.74, and for Facilitating Conditions to α = 0.78. Cronbach α was not calculated for Social Influence, as the scale consisted only of two items.

Main scales of the online survey that had been adapted to the purpose of this study are available in supplementary material 2.

### Data analysis

The focus group sessions were recorded, and notes were taken during and after the discussions. As the primary purpose of the focus groups was to gather initial feedback from the target group, the analysis followed a pragmatic approach in order to directly improve the toolkit. We collected the feedback and listed key points. We prioritized the feedback based on feasibility and urgency.

All scales of the evaluation survey were analysed descriptively with means, standard deviations, ranges, frequencies, and percentages where applicable. We carried out repeated-measures analyses of variance (ANOVAs) to conduct within-subject comparisons of mean scores of the UTAUT concepts Behavioural Intention and Performance Expectancy between the three disorders anorexia nervosa, bulimia nervosa, and binge eating disorder at a significance level of *p* = .05. We examined data characteristics and key assumptions by identifying outliers, conducting Shapiro-Wilk-tests and graphical examinations of normality, and testing the sphericity assumption using Mauchly’s tests. While the normality assumption according to the Shapiro-Wilk tests was not fulfilled, the histograms revealed slightly skewed data approximating normal distributions. We therefore conducted and reported ANOVAs due to their robustness [33]. The effects remained significant with *p* < .001 after applying Greenhouse-Geisser corrections as the assumption of sphericity had been violated according to Mauchly’s tests. Finally, we calculated Pearson correlations to find exploratory associations of selected concepts.

The open-ended remarks of the online-evaluation were collected in an excel file. Two coders conducted a content analysis following a complementary approach that combines deductive and inductive practises [34]. Codes were assigned for the general assessment of the toolkit and for the separate modules. All codes were deductively assigned to the categories “appreciated”, “criticism”, and “wishes” that were in turn divided into subcategories relating to either design aspects or content-related aspects. The other codes were assigned inductively.

## Results

### Part I: focus groups

Participants’ feedback on the first version of the toolkit comprised aspects relating to both, the content and the design of the toolkit. Participants positively evaluated the overall user-friendliness and design (e.g. colour scheme, professionalism) and the quality of the information provided in the toolkit (e.g. engaging and new information, scientific content, perception as valuable resource for research purposes).

As participants also suggested changes with regard to the design (e.g. wish for more dropdown text elements, shorter texts, larger font size, and smaller icons) and the content (e.g. adding information about the interpretation of body-mass index scores, more information about available apps and their clinical utility), the toolkit was adapted accordingly prior to the online survey.

However, the participants also expressed needs, that were not considered feasible and would go beyond the aims of the toolkit. An example came up in group 3: “I would have liked to see information on specific applications, differential indications…” As the toolkit did not aim at advertising, this information could not be provided.

An overview about emerging topics, examples, and the status of implementation is given in Table 2:

**Table 2:** Topics of the participants’ feedback in the focus groups and their implementation

Topic	Examples	Implementation status
Design	Font size, colours, images, length of text	Implemented
Content	Literature, recommendations for apps, diagnostic criteria	Implemented, if feasible
Videos	Voice, length, necessity	Implemented, if feasible
Navigation	Dropdown menus, icons, order of headings	Implemented
Technical issues	Login; browser-specific questions	Implemented

Participants confirmed that they would use and recommend the toolkit for their practical work with patients, as expressed by one participant in group 1: “Good links, good things to get hold of, great. I can also imagine using it, recommending it to others… all the information is compiled.”

### Part II: online survey

#### Self-Assessment of technology skills

Concerning the question when they first used a computer, participants reported a mean age of 9.53 years (SD = 3.19, range: 2–19). The age at first use of the internet was M = 11.79, SD = 3.22, range: 6–22. Private use of internet and social media was reported to be M = 2.24 h per day (SD = 1.79, range: 1–14). The mean average use of internet and social media within the profession was M = 1.85 h per day (SD = 1.96, range: 0–10). The ICT Competence (COMPICT) reached an overall mean of 2.98 (SD = 0.48, range = 1.8-4).

#### E-therapy attitudes (ETAM)

Perceived usefulness was rated with M = 2.75 (SD = 0.62, range = 1-3.86). A total of 5 participants (7.6%) expressed a negative attitude towards the usefulness of e-therapy, 10 (15.2%) a neutral attitude, and 51 (77.3%) a positive one.

Relative Advantage was evaluated with M = 1.08 (SD = 0.54, range = 0-2.5). A negative attitude towards the relative advantage was shown by 51 (77.3%), while 14 (21.2%) showed a neutral and one participant (1.5%) a positive attitude.

The results of the single items are reported in supplementary material 3.

#### UTAUT

The results of the UTAUT-concepts are reported in Table 3. The repeated measures ANOVAs found statistically significant group differences (Behavioural Intention: F(2,130) = 16.17, *p* < .001, η_g_²=0.019; Performance Expectancy: F(2,130) = 10.71, *p* < .001, η_g_²=0.009). Bonferroni-corrected pairwise comparisons demonstrated significant differences between anorexia nervosa and bulimia nervosa (Behavioural Intention: t(65)=-4.07, *p* < .001; Performance Expectancy: t(65)=-3.44, *p* = .003) as well as anorexia nervosa and binge eating disorder (Behavioural Intention: t(65)=-4.26, *p* < .001; Performance Expectancy: t(65)=-3.32, *p* = .004), while bulimia nervosa and binge eating disorder did not differ significantly (Behavioural Intention: t(65) = 1.45, *p* = .45; Performance Expectancy: t(65) = 0.50, *p* = 1.00).

**Table 3:** Results of UTAUT-scores with means (M) and standard deviations (SD)

Concept	Diagnosis	M (SD)	range
Behavioural Intention	Anorexia nervosa	3.39 (1.14)	1–5
	Bulimia nervosa	3.68 (0.99)	1–5
	Binge eating disorder	3.72 (1.00)	1–5
	All	3.60 (1.01)	1–5
Performance Expectancy	Anorexia nervosa	3.37 (1.02)	1–5
	Bulimia nervosa	3.56 (0.91)	1–5
	Binge eating disorder	3.57 (0.93)	1–5
	All	3.50 (0.93)	1–5
Effort Expectancy		3.73 (0.65)	1.75–5
Social Influence		2.66 (0.88)	1–4.5
Facilitating Conditions		3.27 (0.91)	1–5

Note. *N* = 66. Scale from 1–5 (1 = does not apply at all; 5 = fully applies)

#### Exploratory analysis

While age and professional experience (*r* = .86, *p* < .001) as well as Behavioural Intention and Performance Expectancy were statistically significantly correlated (*r* = .78, *p* < .001), no further correlations reached statistical significance, with coefficients ranging from − 0.09 to 0.07. Further details are presented in Table 4.

**Table 4:** Pearson correlations with confidence intervals for selected UTAUT-concepts, age, occupational internet use and professional experience

Variable	1	2	3	4
1. Behavioural Intention				
2. Performance Expectancy	0.78**			
	[0.67, 0.86]			
3. age	− 0.09	− 0.06		
	[-0.32, 0.16]	[-0.30, 0.19]		
4. occupational internet use	0.07	0.03	0.07	
	[-0.18, 0.31]	[-0.21, 0.27]	[-0.17, 0.31]	
5. professional experience	− 0.01	0.01	0.86**	− 0.00
	[-0.25, 0.23]	[-0.24, 0.25]	[0.78, 0.91]	[-0.25, 0.24]

Note. Values in square brackets indicate the 95% confidence interval for each correlation. The confidence interval is a plausible range of population correlations that could have caused the sample correlation (Cumming, 2014). * indicates *p* < .05. ** indicates *p* < .01

#### Evaluation of the toolkit and its modules

The overall impression of the toolkit was positive, with a majority of the 66 participants responding positively to the questions, that the toolkit “provided comprehensive information on digital services in the treatment of EDs“ (M = 4.21, SD = 0.64, range = 2–5). More details are presented in Table 5.

**Table 5:** General evaluation of the toolkit / overall impression (Scale from 1–5 (1 = strongly disagree; 5 = strongly agree))

Item	*N*	M (SD)	range
1. The toolkit provided comprehensive information on digital services in the treatment of eating disorders.	66	4.21 (0.64)	2–5
2. The content of the toolkit is relevant to my clinical work.	66	3.71 (0.86)	2–5
3. The content of the toolkit is up-to-date.	66	4.59 (0.55)	3–5

The evaluation of the modules showed positive results as well. Relevance for clinical work was assessed highest in the module “areas of application” and “eating disorders” (M = 4.09 and M = 4.08 respectively). These two modules also reached the highest values in being too extensive with M = 2.25 and M = 2.47. Comprehensiveness was appreciated in all modules and best in the “glossary” (M = 4.54). No module was found to be too superficial, except “eating disorders” with a medium tendency of M = 2.35. More details are presented in Table 6.

**Table 6:** Evaluation of the modules of the toolkit (Scale from 1–5 (1 = strongly disagree; 5 = strongly agree))

Module	Item	*N*	M (SD)	range
Areas of application	1. The content of this module is relevant to my clinical work.	65	4.09 (0.98)	2–5
	2. The presentation of the content of this module is too extensive.	65	2.25 (1.17)	1–5
	3. The content of this module is presented in a comprehensible way.	63	4.38 (0.68)	2–5
	4. The content of this module is too superficial.	63	1.81 (0.95)	1–5
	5. I liked the videos.	54	4.02 (1.11)	1–5
	6. I liked the informative texts.	63	3.92 (0.94)	1–5
Apps & DiGAs*	1. The content of this module is relevant to my clinical work.	65	3.86 (0.98)	2–5
	2. The presentation of the content of this module is too extensive.	66	2.03 (1.08)	1–5
	3. The content of this module is presented in a comprehensible way.	64	4.30 (0.79)	2–5
	4. The content of this module is too superficial.	66	1.86 (0.91)	1–5
	6. I liked the informative texts.	66	4.14 (0.96)	1–5
Eating disorders	1. The content of this module is relevant to my clinical work.	64	4.08 (0.95)	2–5
	2. The presentation of the content of this module is too extensive.	64	2.47 (1.34)	1–5
	3. The content of this module is presented in a comprehensible way.	58	4.48 (0.63)	3–5
	4. The content of this module is too superficial.	62	2.35 (1.04)	1–5
	6. I liked the informative texts.	60	3.87 (0.93)	2–5
Glossary	1. The content of this module is relevant to my clinical work.	63	3.32 (1.04)	1–5
	2. The presentation of the content of this module is too extensive.	63	1.68 (0.95)	1–5
	3. The content of this module is presented in a comprehensible way.	61	4.54 (0.67)	3–5
	4. The content of this module is too superficial.	63	1.90 (1.06)	1–5
	6. I liked the informative texts.	60	4.07 (1.02)	1–5
Literature	1. The content of this module are relevant to my clinical work.	60	3.55 (1.10)	1–5
	2. The presentation of the content of this module is too extensive.	60	1.83 (1.09)	1–5
	3. The content of this module is presented in a comprehensible way.	60	4.40 (0.74)	3–5
	4. The content of this module is too superficial.	61	1.48 (0.81)	1–5
Tips	1. The content of this module is relevant to my clinical work.	63	3.92 (1.02)	2–5
	2. The presentation of the content of this module is too extensive.	65	2.23 (1.11)	1–5
	3. The content of this module is presented in a comprehensible way.	61	4.34 (0.60)	3–5
	4. The content of this module is too superficial.	66	2.09 (1.13)	1–5
	5. I liked the videos.	56	3.89 (1.06)	1–5
	6. I liked the informative texts.	63	4.00 (0.92)	2–5

Note. *DiGA: Digitale Gesundheitsanwendung (digital health application)

#### Web-CLIC (website - clarity, likeability, informativeness, and credibility)

Website clarity was assessed with M = 5.87 (SD = 0.86, range: 3–7), the likeability with M = 5.39 (SD = 1.04, range: 2.67-7), the informativeness with M = 6.10 (SD = 0.75, range: 3.33-7) and the credibility with M = 6.41 (SD = 0.75, range: 4–7).

#### VisAWI-S (short visual aesthetics of websites Inventory)

The visual aesthetics of the toolkit was evaluated using the VisAWI-S (Short Visual Aesthetics of Websites Inventory) with an average of M = 5.27 points (SD = 1.25, range: 2–7).

#### System usability scale (SUS)

System usability reached M = 78.75 (SD = 13.57, range: 40–100).

### Qualitative results

A total of 47 participants took the opportunity to answer at least one of the open-ended questions that were either related to general or to module specific aspects. By this, 227 comments were collected. The participants appreciated the overall structure of the toolkit and the presentation of the content. Text and video information were evaluated positively. Critical voices addressed the amount of information and the quality of the sound in the video files. Wishes and expectations referred to more specific recommendations of particular applications and their integration into treatment concepts. The navigation of the toolkit was both appreciated and criticised with 5 comments in total.

An overview of the general feedback, the respective codes and examples is given in Table 7.

**Table 7:** Coding system of the content analysis with main codes and first level subcodes

Main code	Subcodes	2nd level Subcodes	Examples
Appreciated			
	design & layout		
		structure; images; general design; text; links; videos	*Overall good selection of categories. Neither too much nor too little information. (ID4532*,* Subcode: structure);* *Very clearly laid out. Easy to understand list of the current study situation (ID4562*,* Subcode: text)*
	content		
		“9 truths"*; applicability; diagnostics; evidence; content in general; relevance for clinical work; videos	*Up-to-date information*,* balanced professional evaluation. (ID4536*,* Subcode: evidence);* *Detailed description of possible applications*,* case examples*,* advantages and disadvantages (ID4560*,* Subcode: content in general)*
Criticism			
	design & layout		
		structure; images; general design; text; links; videos	*Scrolling and long text seems a bit confusing to me. (ID4551*,* Subcode: text); The very unstable sound quality of the videos (ID4535*,* Subcode: videos)*
	content		
		“9 truths"*; applicability; diagnostics; evidence; content in general; relevance for clinical work; videos	*A lot of things seemed “self-evident” to me - wouldn’t have needed some of the content. (ID4669*,* Subcode: content in general); In most places*,* “no clear recommendation” was made for use. In view of the current state of research*,* this is certainly justified*,* but for clinical practice it would of course be nice to have a clear recommendation for action (ID4686*,* Subcode: relevance for clinical work)*
Wishes			
	design & layout		
		structure; images; general design; text; links; videos	*Sample images of what applications look like (e.g.*,* in the test reports) so that you can visualize them better. (ID4562*,* Subcode: images); The text could have been more concise or presented differently. Different fonts and sizes would be an idea (ID4565*,* Subcode: text)*
	content		
		“9 truths"*; applicability; diagnostics; evidence; content in general; relevance for clinical work; videos	*I would also be happy to read the opinions of patients who have been “confronted” with the respective apps plus*,* of course*,* testimonials from therapists. (ID4553*,* Subcode: applicability)* *What are contraindications? (ID4562*,* Subcode: relevance for clinical work)*

Note. *module 9 truths about eating disorders

## Discussion

In this study, a web-based toolkit was developed to inform MHPs about digital interventions for EDs. The toolkit was evaluated in a two-step approach. After a phase of focus groups, adjustments were made. The final version was then evaluated in a cross-sectional online study.

### Assessment of the overall feasibility and acceptability of the toolkit

Overall, MHPs appreciated the toolkit. They gave high ratings for website clarity, visual aesthetics and system usability. After a general evaluation, the specific modules of the toolkit were assessed, while especially the topicality and the comprehensiveness of the content reached high ratings. Participants also recommended some changes which will allow for further improvements of the toolkit. In general, participants gave positive feedback regarding the design, the professional appeal, and the informative value of the toolkit. No user failed to complete the toolkit due to usability issues. The quantitative results show that SUS reached a mean score of M = 78.75, corresponding to a good performance (B+) following the scoring of Sauro-Lewis [29]. Few voices raised in the qualitative comments wished dropdown menus and a direct navigation to specific interventions. However, the latter was not within the intended scope of the toolkit as it does not aim at advertising. The results don’t allow an assessment, if the participants evaluated the toolkit within their clinical routines or at home. Nonetheless, the intended use of the toolkit allows for either getting a broad overview over the topic or targeted answers to specific details that might come up in a stressful working environment.

### Attitudes of MHPs towards digital interventions for the treatment of eds

One of the key findings of this study was that MHPs still report a lack of knowledge and guidance on how to use digital interventions for the benefit of their patients with EDs. In particular, they are unclear about the appropriate settings and tools. Participants in the first part of the study frequently asked for recommendations for specific apps and experiences with them. However, both could not be implemented, as the first wish went beyond the objectives of the toolkit, as it was intended to provide scientific evidence rather than promote individual applications. Moreover, reporting on experiences was not feasible, as still no experiences with reimbursable apps for EDs in Germany have been reported in the scientific literature so far. Another aim of this study was to investigate the intention of MHPs to integrate digital interventions for EDs into routine care. The majority of participants (77%) showed positive general attitudes towards the usefulness of digital interventions for the treatment of EDs as measured by the ETAM. More specifically, the perceived usefulness of digital interventions for EDs was high, while the results for relative advantage remained at a lower level. Relative advantage, in this context, refers to the advantages perceived by MHPs of digital approaches compared to face-to-face therapy. The authors of the ETAM reported similar results. They applied their model to the acceptance of internet-based mental health services in the general population [35]. Higher values for the relative advantage of digital care concepts might be reached for the application of blended care, which was not the aim of this study. Former results show that the acceptance of psychotherapists toward combining face-to-face and digital care approaches is moderate [36], however, the results were not specific to ED treatment.

The results of our study confirm, that the general knowledge about e-mental health applications, which are even reimbursable in the German health system, is still low among psychotherapists and general practitioners [37]. The fact, that only few digital interventions so far target EDs and these few are not suitable for all kinds of ED symptoms [38], further aggravates the situation.

Being asked for the intention to include digital interventions into routine care, MHPs accordingly expressed a medium Behavioural Intention to do so and a medium Performance Expectancy of digital interventions following the UTAUT variables. Both concepts showed a significant correlation in our sample. A similar observation was made in a survey of 118 mental health professionals who treat EDs. On the one hand, they were enthusiastic and endorsed the potential of mental health apps to support their patients, but on the other hand, they remained hesitant to use them on a large scale [18]. The authors explain this discrepancy with insecurity of MHPs regarding the credibility and appropriateness of the apps. Interestingly, for both concepts the values were higher for binge eating disorder and bulimia nervosa, than for anorexia nervosa. This corresponds to former results, where MHPs expressed doubts in using digital interventions for individuals with anorexia nervosa [10]. Also other authors have argued earlier that the severity of this disease requires a face-to-face contact [39]. Due to the complexity of the disorder, its medical complications and the multitude of comorbidities [40], digital interventions for patients with anorexia nervosa must be carefully evaluated by treating physicians. It is important to note, that the therapeutic alliance impacts the outcomes of therapy especially in adults with anorexia nervosa [41], which is limited in the use of a digital intervention, even in a blended concept. However, the applicability of digitally delivered post-acute care, have been appreciated by MHPs [42].

### Strengths and limitations

To the best of our knowledge this is the first study that developed and evaluated an information platform for digital interventions in the treatment of EDs. It was a strength of the study concept to iteratively develop and assess the toolkit. This had the advantage of being able to directly integrate MHPs feedback and continuously enhance the quality of the website. However, a bigger sample size with a representative sample of different professional groups (e.g., psychiatrists and other medical doctors) would have allowed to carry out more targeted analyses, for example exploratory subgroup analyses. The current version of the toolkit is addressed to both specialist groups, MHPs for adults as well as children and adolescents. Though the majority of our respondents had a psychological background, recent literature has shown that physicians as well show openness towards the use of digital application in mental health care, however, results specific to EDs are missing so far [43]. In accordance with our findings, training needs of physicians regarding the prescription of DiGAs have been reported as well [44]. To conclude, future studies investigating medical mental health professionals in particular might reach results comparable to our findings. Moreover, our sample might show a gender bias with 87.9% female participants. However, this high proportion is not too far from the actual gender distribution of mental health professionals working in Germany, as the Association of Statutory Health Insurance Physicians estimates that women accounted for 77.2% of German psychotherapists and 67.1% of medical doctors working as psychotherapists in 2023 [45]. Another limitation is that the duration of use of the app was not recorded during the online survey, which limits the assessment of actual usage. We decided against recording this information because MHPs can easily be distracted by their clinical routines (e.g., phone calls) and the duration of use would not have led to meaningful assessments.

A final limitation concerns the recruitment strategy that has been planned by means of convenience sampling. This approach is driven by the accessibility of the study population to the research team, but might result in reduced external validity and thus, limited generalizability [46].

### Future research

Considering the constantly growing evidence-base for digital interventions in the ED treatment, future versions of the toolkit have to be released and evaluated again. The content is made available upon request for translation into other languages to extend the toolkits’ reach and broader use. With greater integration of digital interventions into routine care, case reports with treatment recommendations could also be made available. Besides from more nuanced and targeted tools in ED treatment, new developments, e.g., those using artificial intelligence will pose further challenges on the task of informing MHPs about benefits and challenges [47].

However, investigating usability and acceptance is only a first step to provide guidance for MHPs. Following Kirkpatrick’s model of 4 levels of evaluation, our study remained on level 1, i.e. reaction. Learning effectiveness, behavioural outcomes and long-term results (levels 2–4) build on this first level [48]. The next step should focus on examining the effectiveness of the toolkit and its impact on clinical routines. To achieve this, an analysis of the increase in knowledge and competence among a representative sample of relevant stakeholders would be necessary.

## Conclusions

Though MHPs show positive attitudes towards the use of digital health for patients with EDs, their knowledge and experiences in this area are still limited. The web-based toolkit is a viable way to meet their needs. Future developments of more specific digital interventions targeted at ED treatment in different treatment settings and for different diagnoses will add on the existing knowledge and provide a clearer evidence base. With a growing body of literature reporting experiences with specific applications in the future, a more targeted information need can be met. Finally, with a future version, the effectiveness of the toolkit regarding reach and learning success for MHPs in different disciplines can be tested.

## Supplementary Information

Below is the link to the electronic supplementary material.

Supplementary Material 1

Supplementary Material 2

Supplementary Material 3

## Data Availability

The datasets used and/or analysed during the current study are available from the corresponding author on reasonable request.

## Abbreviations

DiGA: Digitale Gesundheitsanwendung (digital health application)
ED: Eating disorder
ETAM: E-Therapy Attitudes
MHPs: Mental health professionals
SUS: System Usability Scale
UTAUT: Unified Theory of Acceptance and Use of Technology
VisAWI-S: Short Visual Aesthetics of Websites Inventory
Web-CLIC: Website - Clarity, Likeability, Informativeness, and Credibility
