# Supplementary material for: Development and evaluation of a web-based toolkit to inform mental health professionals about digital mental health interventions for eating disorders
Source: J Eat Disord. 2026 Jan 9;14:33. doi: 10.1186/s40337-025-01518-1 (PMC12849147; doi:10.1186/s40337-025-01518-1)
Supplement: Supplementary file 1 — Supplementary Material 1 [file 40337_2025_1518_MOESM1_ESM.docx]

# Supplementary Material I: Elements of the Toolkit

The following elements can be accessed at: <https://www.sida-essstoerungen.de/sidaess/toolkit>

| Menu / Module | Original (German) | Description / Subheadings | Format |
| --- | --- | --- | --- |
| Home | Home | Welcome; News; general description of the purpose of the toolkit | Text, Video |
| Eating Disorders | Essstörungen | The most important facts in brief; Diagnostic Criteria (ICD-10); “9 truths about EDs”; Prevalences (international); Information on BMI; Prognosis; EDs and digital services; References | Text |
| Tips | Tipps | The most important facts in brief; 5 recommendations for the use of digital applications in the treatment of EDs; References | Text, Video |
| Digital Health Applications | DiGAs | The most important facts in brief; DiGAs and DiGA-Index; M-Health index & Navigation Database; References | Text |
| Areas of Application | Anwendungsbereiche | Areas of application; Prevention and health promotion; Self-management and self-help; Therapy-accompanying services (blended care); Aftercare and relapse prevention; Digital services for parents and relatives; Online counselling | Text, Videos in Submenues |
| Glossary | Glossar | Central terminology at a glance | Text |
| Literature | Literatur | Link to current Pubmed Search with search strings; Literature list sorted by areas of application | Text |
| Contact | Kontakt | Contact information | Text and form |

Examples of screenshots:


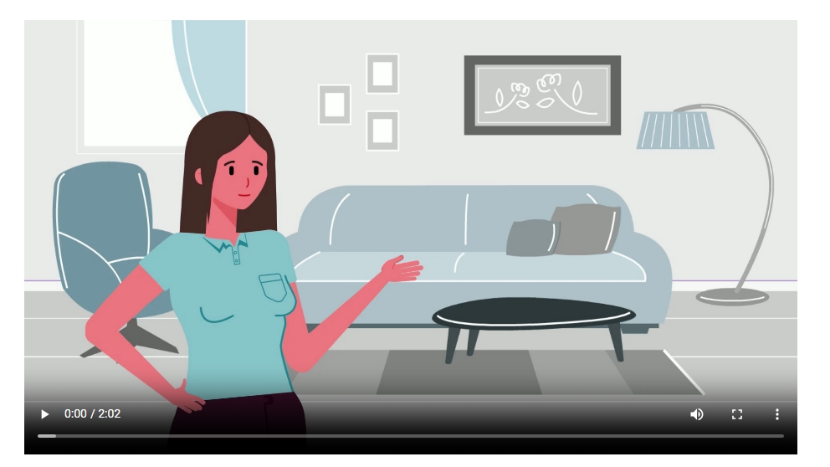


Figure 1: example video (blended care)


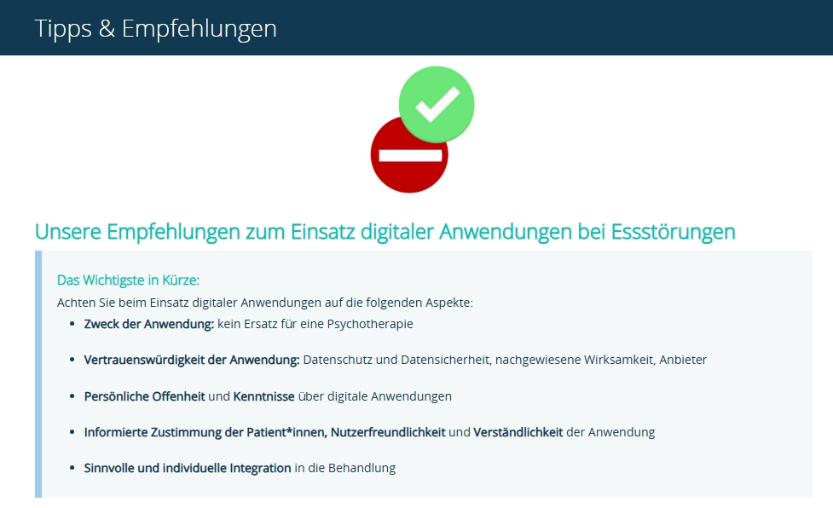


Figure 2: Recommendations for the use of digital application in the treatment of EDs

Translation of the text in figure 2:

The most important facts in brief:

- When using digital applications, pay attention to the following aspects:
- Purpose of the application: not a substitute for psychotherapy
- Trustworthiness of the application: data protection and data security, proven effectiveness, provider
- Personal openness and knowledge of digital applications
- Informed consent of patients, user-friendliness and comprehensibility of the application
- Meaningful and individual integration into treatment
