# Supplementary material for: Development and evaluation of a web-based toolkit to inform mental health professionals about digital mental health interventions for eating disorders
Source: J Eat Disord. 2026 Jan 9;14:33. doi: 10.1186/s40337-025-01518-1 (PMC12849147; doi:10.1186/s40337-025-01518-1)
Supplement: Supplementary file 2 — Supplementary Material 2 [file 40337_2025_1518_MOESM2_ESM.docx]

# Supplementary Material II: Online-Questionnaire (Toolkit-Evaluation)

# *(see English translations p. 10 f.)*

## Allgemeine Toolkit-Evaluation/ Gesamteindruck

Bitte nehmen Sie sich nun einen Augenblick Zeit, um die folgenden Fragen zum Toolkit zu beantworten:

Bitte geben Sie auf einer Skala von 1-5 (1= stimme überhaupt nicht zu; 5 = stimme voll zu) an, wie Sie das Toolkit insgesamt beurteilen.

| **Nr.** | **Item** | **Antwort** |
| --- | --- | --- |
| 1 | Ich wurde durch das Toolkit umfassend zu digitalen Angeboten in der Essstörungsbehandlung informiert. | 5-stufige Skala |
| 2 | Die Inhalte des Toolkits sind für meine klinische Tätigkeit relevant. | 5-stufige Skala |
| 3 | Die Inhalte des Toolkits sind aktuell. | 5-stufige Skala |
| 4 | Ich habe folgende Inhalte vermisst (optional): | [freies Textfeld] |
| 5 | Mir haben folgende Inhalte besonders gut gefallen (optional): | [freies Textfeld] |
| 6 | Mich haben folgende Inhalte gestört (optional): | [freies Textfeld] |

## Web-CLIC (Clarity, Likeability, Informativeness, and Credibility)^[[1]](#footnote-1)^

Im folgenden Fragebogen möchten wir Sie bitten, die Inhalte und Darstellung des Toolkits zu bewerten. Bitte beurteilen Sie den Inhalt des Toolkits insgesamt anhand der folgenden Aussagen auf einer Skala von 1 (stimme gar nicht zu) bis 7 (stimme voll zu). Vielen Dank!

| **Nr.** | **Item** | **Antwort** |
| --- | --- | --- |
| 1 | Die Inhalte sind anschaulich aufbereitet. | 7-stufige Skala  1 = stimme gar nicht zu  2 = stimme nicht zu  3 = stimme eher nicht zu  4 = neutral  5 = stimme eher zu  6 = stimme zu  7 = stimme voll zu |
| 2 | Die Texte liefern mir kurz und bündig die wichtigsten Informationen. | 7-stufige Skala |
| 3 | Der Sprachgebrauch in den Texten ist geläufig und allgemein verständlich. | 7-stufige Skala |
| 4 | Das Toolkit weckt mein Interesse. | 7-stufige Skala |
| 5 | Die Inhalte des Toolkits sind spannend. | 7-stufige Skala |
| 6 | Ich lese die Texte des Toolkits gerne. | 7-stufige Skala |
| 7 | Die Informationen sind qualitativ hochwertig. | 7-stufige Skala |
| 8 | Ich finde, die Informationen in dem Toolkit sind nützlich. | 7-stufige Skala |
| 9 | Das Toolkit ist informativ. | 7-stufige Skala |
| 10 | Die in dem Toolkit dargebotenen Informationen sind glaubwürdig. | 7-stufige Skala |
| 11 | Die in dem Toolkit dargebotenen Informationen sind seriös. | 7-stufige Skala |
| 12 | Ich kann den Informationen in dem Toolkit vertrauen. | 7-stufige Skala |

## VisAWI-S (Short Visual Aesthetics of Websites Inventory)^[[2]](#footnote-2)^

Bitte beurteilen Sie auf einer Skala von 1 (stimme gar nicht zu) bis 7 (stimme voll zu), inwieweit Sie den folgenden Aussagen in Bezug auf das Toolkit zustimmen. Vielen Dank!

| **Nr.** | **Item** | **Antwort** |
| --- | --- | --- |
| 1 | Auf der Toolkit-Seite passt alles zusammen. | 7-stufige Skala  1 = stimme gar nicht zu  2 = stimme nicht zu  3 = stimme eher nicht zu  4 = neutral  5 = stimme eher zu  6 = stimme zu  7 = stimme voll zu |
| 2 | Das Layout ist angenehm vielseitig. | 7-stufige Skala |
| 3 | Die farbliche Gesamtgestaltung wirkt attraktiv. | 7-stufige Skala |
| 4 | Das Layout ist professionell. | 7-stufige Skala |

## SUS (System Usability Scale)^[[3]](#footnote-3)^

Im nächsten Fragebogen geht es um die Bewertung der so genannten Usability bzw. Bedienerfreundlichkeit. Sie haben die Möglichkeit einzelne Aspekte auf einer Skala von 1 (Stimme überhaupt nicht zu) bis 5 (Stimme voll zu) zu bewerten. Vielen Dank!

| **Nr.** | **Item** | **Antwort** |
| --- | --- | --- |
| 1 | Ich denke, dass ich das Toolkit gerne häufig benutzen würde. | 5-stufige Skala |
| 2 | Ich fand das Toolkit unnötig komplex. | 5-stufige Skala |
| 3 | Ich fand das Toolkit einfach zu benutzen. | 5-stufige Skala |
| 4 | Ich glaube, ich würde die Hilfe einer technisch versierten Person benötigen, um das Toolkit benutzen zu können. | 5-stufige Skala |
| 5 | Ich fand, die verschiedenen Funktionen in diesem Toolkit waren gut integriert. | 5-stufige Skala |
| 6 | Ich denke, das Toolkit enthielt zu viele Inkonsistenzen. | 5-stufige Skala |
| 7 | Ich kann mir vorstellen, dass die meisten Menschen den Umgang mit diesem Toolkit sehr schnell lernen. | 5-stufige Skala |
| 8 | Ich fand das Toolkit sehr umständlich zu nutzen. | 5-stufige Skala |
| 9 | Ich fühlte mich bei der Benutzung des Toolkits sehr sicher. | 5-stufige Skala |
| 10 | Ich musste eine Menge lernen, bevor ich anfangen konnte, das Toolkit zu verwenden. | 5-stufige Skala |

## Modulspezifische Evaluation

Im Folgenden möchten wir Ihnen Fragen zu den einzelnen Modulen des Toolkits stellen. *(Die hier aufgeführten Fragen werden für jedes Modul des Toolkits separat gestellt)*

**Modul x ____________________** *(Titel wird ergänzt)*

Bitte geben Sie auf einer Skala von 1-5 (1= stimme überhaupt nicht zu; 5 = stimme voll zu) an, wie Sie das Modul beurteilen. Wählen Sie bei Bedarf das Kontrollkästchen aus, um weitere Informationen über das Modul zu erhalten.

| **Nr.** | **Item** | **Antwort** |
| --- | --- | --- |
| 1 | Die Inhalte des Moduls sind für meine klinische Tätigkeit relevant. | 5-stufige Skala |
| 2 | Die Darstellung der Inhalte dieses Moduls ist zu umfangreich. | 5-stufige Skala |
| 3 | Die Inhalte dieses Moduls sind verständlich aufbereitet. | 5-stufige Skala |
| 4 | Die Inhalte dieses Moduls sind zu oberflächlich. | 5-stufige Skala |
| 5 | Die Videos haben mir gut gefallen. | 5-stufige Skala |
| 6 | Die Informationstexte haben mir gut gefallen. | 5-stufige Skala |
| 7 | Ich habe folgende Inhalte vermisst *(optional)*: | [freies Textfeld] |
| 8 | Mir haben folgende Inhalte besonders gut gefallen *(optional)*: | [freies Textfeld] |
| 9 | Mich haben folgende Inhalte gestört *(optional)*: | [freies Textfeld] |

## Allgemeine Einstellungen zu digitalen Angeboten

(nach ETAM, E-Therapy Attitude Scales^[[4]](#footnote-4)^)

Im Folgenden möchten wir Sie zu Ihren Erfahrungen mit digitalen Angeboten bzw. digitalen Interventionen befragen. Dabei geht es um Angebote, die folgende Komponenten enthalten können:

- Online-Beratung (E-Mail, Chat)
- Mobile (Smartphone-)Apps
- Fitnessarmbänder, Wearables
- Therapeutische, browserbasierte Programme, online-Selbsthilfeprogramme
- Interventionen aus dem Bereich der Augmented/Virtual Reality
- Andere (z.B. Biofeedback, Spiele)

Mit digitalen Angeboten bzw. digitalen Interventionen ist ***keine*** Fernbehandlung mittels Videokonferenz gemeint.

Bitte geben Sie bei den folgenden Aussagen zu digitalen Interventionen spontan an, inwieweit Sie ihnen zustimmen oder nicht zustimmen - von 1 „stimmt nicht“ (=stimme überhaupt nicht zu) bis 5 „stimmt genau“ (=stimme völlig zu). Es geht dabei um Ihre persönliche, aktuelle Meinung.

| **Nr.** | **Item** | **Antwort** |
| --- | --- | --- |
| 1 | ^8^Digitale Interventionen sind modern bzw. entsprechen unserer heutigen Zeit. | 5-stufige Skala |
| 2 | ^8^Digitale Interventionen werden konventionelle Psychotherapien zukünftig ersetzen können. | 5-stufige Skala |
| 3 | ^8^Digitale Interventionen lassen sich besser mit Arbeit und Privatleben vereinbaren als konventionelle Psychotherapien. | 5-stufige Skala |
| 4 | ^8^Es macht für mich keinen Unterschied, ob eine Psychotherapie digital oder in der Praxis erfolgt. | 5-stufige Skala |
| 5 | ^8^Digitale Interventionen werden mehr Personen mit psychischen Problemen erreichen. | 5-stufige Skala |
| 6 | ^8^Krankenkassen sollten die Kosten für digitale Interventionen übernehmen. | 5-stufige Skala |
| 7 | ^8^Digitale Interventionen sind vergleichbar wirksam wie konventionelle Psychotherapien. | 5-stufige Skala |
| 8 | ^8^Das Vertrauen zu einer*m Therapeut*in kann digital genauso gut aufgebaut werden wie bei konventionellen Psychotherapien. | 5-stufige Skala |
| 9 | ^8^Digitale Interventionen sind eine geeignete Alternative zu konventionellen Psychotherapien. | 5-stufige Skala |
| 10 | ^8^Bei psychischen Problemen würde ich eine digitale Intervention in Anspruch nehmen. | 5-stufige Skala |
| 11 | ^8^Ich würde eine digitale Intervention einer konventionellen Psychotherapie vorziehen. | 5-stufige Skala |
| 12 | ^8^Digitale Interventionen werden mehr Patient*innen erreichen und ihnen helfen können. | 5-stufige Skala |
| 13 | ^8^Ich mache mir keine besonderen Sorgen um den Datenschutz bei digitalen Angeboten. | 5-stufige Skala |
| 14 | ^8^Durch die Anonymität bei digitalen Interventionen sinkt die Hemmschwelle, offen und ehrlich über wichtige Probleme zu sprechen. | 5-stufige Skala |
| 15 | ^8^Durch die Verbreitung von digitalen Interventionen werden sich Menschen früher professionelle Hilfe holen. | 5-stufige Skala |
| 16 | ^8^Missverständnisse treten bei digitalen Interventionen ähnlich häufig auf wie bei konventionellen Psychotherapien. | 5-stufige Skala |
| 17 | ^8^Digitale Interventionen eignen sich für die meisten Patient*innen, unabhängig vom persönlichen Hintergrund (Alter, Geschlecht, Bildung, etc.). | 5-stufige Skala |

## Einstellungen zu störungsspezifischen digitalen Angeboten

Nach UTAUT (Unified Theory of Acceptance and Use of Technology )^[[5]](#footnote-5)^

Im Folgenden möchten wir Sie zu Ihren Erfahrungen mit digitalen Angeboten bzw. digitalen Interventionen in der Behandlung **von Essstörungen** befragen.

Dabei geht es um Angebote, die folgende Komponenten enthalten können:

- Online-Beratung (E-Mail, Chat)
- Mobile (Smartphone-)Apps
- Fitnessarmbänder, Wearables
- Therapeutische, browserbasierte Programme, online-Selbsthilfeprogramme
- Interventionen aus dem Bereich der Augmented/Virtual Reality
- Andere (z.B. Biofeedback, Spiele)

Mit digitalen Angeboten bzw. digitalen Interventionen ist ***keine*** Fernbehandlung mittels Videokonferenz gemeint.

| **Konstrukt** | **Nr.** | **Item** | **Antwort** |
| --- | --- | --- | --- |
| Behavioral Intention | 1 | ²Ich könnte mir vorstellen, bei meiner Arbeit mit Betroffenen eine digitale Intervention auszuprobieren.  o Anorexie  o Bulimie  o Binge-Eating Störung | 5-stufige Skala  1 stimmt nicht  5 stimmt genau |
|  | 2 | ²Wenn man es mir anbieten würde, würde ich regelmäßig eine digitale Intervention bei meiner Arbeit mit Betroffenen nutzen.  o Anorexie  o Bulimie  o Binge-Eating Störung | 5-stufige Skala |
|  | 3 | **²**Ich würde meinen Fachkolleg*innen eine digitale Intervention für die Arbeit mit Betroffenen empfehlen.  o Anorexie  o Bulimie  o Binge-Eating Störung | 5-stufige Skala |
| Perfor-mance Expectancy | 4 | ²Die Nutzung einer digitalen Intervention würde die Wirksamkeit meiner therapeutischen Arbeit erhöhen.  o Anorexie  o Bulimie  o Binge-Eating Störung | 5-stufige Skala |
|  | 5 | **²**Die Nutzung einer digitalen Intervention wäre förderlich für die Gesundheit von Betroffenen.  o Anorexie  o Bulimie  o Binge-Eating Störung | 5-stufige Skala |
|  | 6 | ²Insgesamt würde eine digitale Intervention Betroffene dabei unterstützen, mit ihren Problemen umzugehen.  o Anorexie  o Bulimie  o Binge-Eating Störung | 5-stufige Skala |

Beachten Sie, dass sich die folgenden Fragen auf die Behandlung **von Essstörungen** beziehen.

| Effort Expectancy | 7 | ²Für **Behandelnde** wäre es einfach, eine digitale Intervention zu nutzen. | 5-stufige Skala  1 stimmt nicht  5 stimmt genau |
| --- | --- | --- | --- |
|  | 8 | ²Für **Betroffene** wäre es einfach, eine digitale Intervention zu nutzen. | 5-stufige Skala |
|  | 9 | ²Eine digitale Intervention wäre für **mich** klar und leicht zu verstehen. | 5-stufige Skala |
|  | 10 | ²Eine digitale Intervention wäre für **Betroffene** klar und leicht zu verstehen. | 5-stufige Skala |
| Social Influence | 11 | ²Meine Kolleg*innen würden mir empfehlen, eine digitale Intervention für meine therapeutische Arbeit zu nutzen. | 5-stufige Skala |
|  | 12 | ²Betroffene würden mich in meiner Arbeit darauf hinweisen, eine digitale Intervention zu nutzen. | 5-stufige Skala |
| Facilitating Conditions | 13 | ²Bei **mir** sind alle technischen Voraussetzungen erfüllt, um eine digitale Intervention in meiner Arbeit zu nutzen. | 5-stufige Skala |
|  | 14 | ²Bei **Betroffenen** sind alle technischen Voraussetzungen erfüllt, um eine digitale Intervention zu nutzen. | 5-stufige Skala |
|  | 15 | ²Wenn **ich** technische Probleme mit einer digitalen Intervention hätte, würde ich technische Unterstützung erhalten. | 5-stufige Skala |
|  | 16 | ²Wenn **Betroffene** technische Probleme mit einer digitalen Intervention hätten, würden sie technische Unterstützung erhalten. | 5-stufige Skala |

# English Translations

## General Evaluation of the Toolkit-Evaluation/ overall impression

Please take a moment to answer the following questions about the toolkit:

Please rate your overall assessment of the toolkit on a scale of 1-5 (1 = strongly disagree; 5 = strongly agree).

| **Nr.** | **Item** | **response** |
| --- | --- | --- |
| 1 | The toolkit provided comprehensive information on digital services in the treatment of eating disorders. | 5-point scale |
| 2 | The content of the toolkit is relevant to my clinical work. | 5-point scale |
| 3 | The content of the toolkit is up-to-date. | 5-point scale |
| 4 | I missed the following content (optional): | [open text] |
| 5 | I particularly liked the following content (optional): | [open text] |
| 6 | The following content bothered me (optional): | [open text] |

## VisAWI-S (Short Visual Aesthetics of Websites Inventory) ^[[6]](#footnote-6)^

Please rate on a scale of 1 (strongly disagree) to 7 (strongly agree) the extent to which you agree with the following statements regarding the toolkit. Thank you very much!

| **Nr.** | **Item** | **Response** |
| --- | --- | --- |
| 1 | Everything goes together on this site.* | 7-point scale  1 = strongly disagree  2 = disagree  3 = somewhat disagree  4 = neutral  5 = somewhat agree  6 = agree  7 = strongly agree |
| 2 | The layout is pleasantly varied. | 7-point scale |
| 3 | The color composition is attractive. | 7-point scale |
| 4 | The layout appears professionally designed. | 7-point scale |

## Web-CLIC (Clarity, Likeability, Informativeness, and Credibility)^[[7]](#footnote-7)^

In the following questionnaire, we would like to ask you to evaluate the content and presentation of the toolkit. Please rate the overall content of the toolkit based on the following statements on a scale from 1 (strongly disagree) to 7 (strongly agree). Thank you very much!

| **Nr.** | **Item** | **Response** |
| --- | --- | --- |
| 1 | The contents of the toolkit are clearly presented.* | 7-point scale  1 = strongly disagree  2 = disagree  3 = somewhat disagree  4 = neutral  5 = somewhat agree  6 = agree  7 = strongly agree |
| 2 | The texts provide me information in a clear and concise manner. | 7-point scale |
| 3 | The language used in the texts is current and easy to understand. | 7-point scale |
| 4 | The toolkit arouses my interest. | 7-point scale |
| 5 | The contents of the toolkit are exciting. | 7-point scale |
| 6 | I enjoy reading the toolkit. | 7-point scale |
| 7 | The information is of high quality. | 7-point scale |
| 8 | I find the information in the toolkit to be useful. | 7-point scale |
| 9 | The toolkit is informative. | 7-point scale |
| 10 | I find the information provided on the toolkit to be authentic. | 7-point scale |
| 11 | The information provided in the toolkit is reliable. | 7-point scale |
| 12 | I can trust the information in the toolkit. | 7-point scale |

## SUS (System Usability Scale)^[[8]](#footnote-8)^

The next questionnaire concerns the evaluation of usability or user-friendliness. You have the opportunity to rate individual aspects on a scale from 1 (strongly disagree) to 5 (strongly agree). Thank you very much!

| **Nr.** | **Item** | **Response** |
| --- | --- | --- |
| 1 | I think that I would like to use this toolkit frequently.* | 5-point scale |
| 2 | I found the toolkit unnecessarily complex. | 5-point scale |
| 3 | I thought the toolkit was easy to use. | 5-point scale |
| 4 | I think that I would need the support of a technical person to be able to use this toolkit. | 5-point scale |
| 5 | I found the various functions in this toolkit were well integrated. | 5-point scale |
| 6 | I thought there was too much inconsistency in this toolkit. | 5-point scale |
| 7 | I would imagine that most people would learn to use this toolkit very quickly. | 5-point scale |
| 8 | I found the toolkit very cumbersome to use. | 5-point scale |
| 9 | I felt very confident using the toolkit. | 5-point scale |
| 10 | I needed to learn a lot of things before I could get going with this toolkit. | 5-point scale |

## Evaluation of single modules

Below, we would like to ask you some questions about the individual modules of the toolkit. *(The questions listed here are asked separately for each module of the toolkit.)*

**Modul x ____________________** *(Title to be added)*

Please rate the module on a scale of 1-5 (1 = strongly disagree; 5 = strongly agree). If necessary, select the checkbox to receive further information about the module.

| **Nr.** | **Item** | **Antwort** |
| --- | --- | --- |
| 1 | The content of this module is relevant to my clinical work. | 5-point scale |
| 2 | The presentation of the content of this module is too extensive. | 5-point scale |
| 3 | The content of this module is presented in a comprehensible way. | 5-point scale |
| 4 | The content of this module is too superficial. | 5-point scale |
| 5 | I liked the videos. | 5-point scale |
| 6 | I liked the informative texts. | 5-point scale |
| 7 | I missed the following content (optional): | [open text] |
| 8 | I particularly liked the following content (optional): | [open text] |
| 9 | The following content bothered me (optional): | [open text] |

## General attitudes towards digital interventions

(derived from ETAM, E-Therapy Attitude Scales^[[9]](#footnote-9)^)

In the following, we would like to ask you about your experiences with digital services and digital interventions. These are services that may include the following components:

- Online counselling (e-mail, chat)
- Mobile (smartphone) apps
- Fitness wristbands, wearables
- Therapeutic, browser-based programmes, online self-help programmes
- Interventions in the field of augmented/virtual reality
- Other (e.g. biofeedback, games)

Digital services or digital interventions do not refer to remote treatment via video conferencing.

Please spontaneously indicate to what extent you agree or disagree with the following statements on digital interventions – from 1 ‘disagree’ (=strongly disagree) to 5 ‘agree’ (=strongly agree). This is concerning your personal, current opinion.

| **Nr.** | **Item** | **response** |
| --- | --- | --- |
| 1 | Digital interventions* are modern and in line with our modern times. | 5-point scale |
| 2 | Digital interventions will replace conventional face-to-face psychotherapy in the future. | 5-point scale |
| 3 | Digital interventions are more compatible with work and private life than conventional face-to-face therapy. | 5-point scale |
| 4 | It makes no difference to me whether psychotherapy is conducted through the internet or in a psychotherapy practice in a clinic. | 5-point scale |
| 5 | Digital interventions will reach more individuals with mental health problems. | 5-point scale |
| 6 | Health insurance companies should cover the costs for digital interventions. | 5-point scale |
| 7 | Digital interventions are as effective as conventional face-to-face psychotherapies. | 5-point scale |
| 8 | Trust in a therapist can be just as easily built on the internet as in conventional face-to-face psychotherapy. | 5-point scale |
| 9 | Digital interventions are an appropriate alternative to conventional face-to-face psychotherapy. | 5-point scale |
| 10 | In case of mental health problems, I would attend a digital intervention. | 5-point scale |
| 11 | I would prefer a digital intervention to a conventional face-to-face psychotherapy. | 5-point scale |
| 12 | Digital interventions will reach more patients and help them. | 5-point scale |
| 13 | I’m not particularly worried about data security in digital interventions. | 5-point scale |
| 14 | The anonymity in digital interventions decreases the threshold to speak openly and honestly about important issues. | 5-point scale |
| 15 | Through the dissemination of digital interventions, persons will get professional help earlier. | 5-point scale |
| 16 | Misunderstandings occur in digital interventions as often as in conventional psychotherapies. | 5-point scale |
| 17 | Digital interventions are suitable for most patients, regardless of their personal background (age, sex, education, etc). | 5-point scale |

**Attitudes towards disorder-specific digital services**

Derived from UTAUT (Unified Theory of Acceptance and Use of Technology )^[[10]](#footnote-10)^

In the following, we would like to ask you about your experiences with digital services or digital interventions in the treatment of eating disorders.

These services may include the following components:

- Online counselling (e-mail, chat)
- Mobile (smartphone) apps
- Fitness trackers, wearables
- Therapeutic, browser-based programmes, online self-help programmes
- Interventions in the field of augmented/virtual reality
- Other (e.g. biofeedback, games)

Digital services or digital interventions do **not** refer to remote treatment via video conferencing.

| **Construct** | **Nr.** | **Item** | **response** |
| --- | --- | --- | --- |
| Behavioral Intention | 1 | I could imagine to try out an internet-based intervention in my work with patients*  o Anorexia Nervosa  o Bulimia Nervosa  o Binge-Eating Disorder | 5-point scale  1 Not true  5 Completely true |
|  | 2 | If offered, I would use a digital intervention regularly in my work with patients  o Anorexia Nervosa  o Bulimia Nervosa  o Binge-Eating Disorder | 5-point scale |
|  | 3 | I would recommend a digital intervention to a colleague** for the work with patients  o Anorexia Nervosa  o Bulimia Nervosa  o Binge-Eating Disorder | 5-point scale |
| Perfor-mance Expectancy | 4 | Using a digital intervention would increase the effectiveness of the treatment of my patients.  o Anorexia Nervosa  o Bulimia Nervosa  o Binge-Eating Disorder | 5-point scale |
|  | 5 | Using a digital intervention would be beneficial for the health of my patients.  o Anorexia Nervosa  o Bulimia Nervosa  o Binge-Eating Disorder | 5-point scale |
|  | 6 | Overall, digital intervention would support patients in coping with their problems.  o Anorexia Nervosa  o Bulimia Nervosa  o Binge-Eating Disorder | 5-point scale |

Please note that the following questions relate to the **treatment of eating disorders**.

| Effort Expectancy | 7 | Using a digital intervention would be simple **for mental health professionals.** | 5-point scale  1 Not true  5 Completely true |
| --- | --- | --- | --- |
|  | 8 | Using a digital intervention would be simple **for patients.** | 5-point scale |
|  | 9 | A digital intervention would be clear and easily comprehensible **to me.** | 5-point scale |
|  | 10 | A digital intervention would be clear and easily comprehensible **to** **patients.** | 5-point scale |
| Social Influence | 11 | My colleagues would recommend me to use a digital intervention in my therapeutic work. | 5-point scale |
|  | 12 | My patients would recommend me to use a digital intervention in my therapeutic work. | 5-point scale |
| Facilitating Conditions | 13 | **I** have all necessary technical preconditions for using a digital intervention in my work. | 5-point scale |
|  | 14 | **Patients** have all necessary technical preconditions for using a digital intervention. | 5-point scale |
|  | 15 | In case of technical problems with a digital intervention **I** would receive technical support. | 5-point scale |
|  | 16 | In case of technical problems with a digital intervention **my patients** would receive technical support. | 5-point scale |

1. Thielsch, M. T. & Hirschfeld, G. (2019). Facets of website content. Human-Computer Interaction, 34 (4), 279-327. [↑](#footnote-ref-1)
2. Moshagen, M. & Thielsch, M. T. (2013). A short version of the visual aesthetics of websites inventory. Behaviour & Information Technology, 32(12), 1305-1311. [↑](#footnote-ref-2)
3. Rummel, B. (2016). System Usability Scale – Jetzt auch auf Deutsch. Zuletzt abgerufen am 07.02.2023 unter <https://blogs.sap.com/2016/02/01/system-usability-scale-jetzt-auch-auf-deutsch/> [↑](#footnote-ref-3)
4. E-therapy attitudes. (Apolinário-Hagen J, Harrer M, Kählke F, Fritsche L, Salewski C, Ebert DD. Public Attitudes Toward Guided Internet-Based Therapies: Web-Based Survey Study. JMIR Ment Health 2018;5(2): e10735. DOI: [10.2196/10735](http://doi.org/10.2196/10735)) [↑](#footnote-ref-4)
5. Philippi, P., Baumeister, H., Apolinário-Hagen, J., Ebert, D. D., Hennemann, S., Kott, L., . . . Terhorst, Y. (2021). Acceptance towards digital health interventions - Model validation and further development of the Unified Theory of Acceptance and Use of Technology. *Internet Interv*, 26, 100459. doi:10.1016/j.invent.2021.100459 [↑](#footnote-ref-5)
6. Moshagen, M. & Thielsch, M. T. (2013). A short version of the visual aesthetics of websites inventory. Behaviour & Information Technology, 32 (12), 1305-1311.

   *Different from the original version, the term „site” has been replaced by “toolkit” in this questionnaire. [↑](#footnote-ref-6)
7. Thielsch MT, Hirschfeld G: Facets of website content. Human–Computer Interaction 2019, 34(4):279-327.

   * The term “website” has been replaced by “toolkit” in this questionnaire. [↑](#footnote-ref-7)
8. Brooke, J. (1996). SUS-A quick and dirty usability scale. Usability evaluation in industry, 189(194), 4-7.
   *Different from the original version, the term “system” has been replaced by “toolkit” in this questionnaire. [↑](#footnote-ref-8)
9. E-therapy attitudes. (Apolinário-Hagen J, Harrer M, Kählke F, Fritsche L, Salewski C, Ebert DD. Public Attitudes Toward Guided Internet-Based Therapies: Web-Based Survey Study. JMIR Ment Health 2018;5(2): e10735. DOI: [10.2196/10735](http://doi.org/10.2196/10735))

   *Different from the original version, the terms „Internet-based therapies“ have been replaced by „digital interventions“ in this questionnaire. [↑](#footnote-ref-9)
10. Philippi, P., Baumeister, H., Apolinário-Hagen, J., Ebert, D. D., Hennemann, S., Kott, L., . . . Terhorst, Y. (2021). Acceptance towards digital health interventions - Model validation and further development of the Unified Theory of Acceptance and Use of Technology. *Internet Interv*, 26, 100459. doi:10.1016/j.invent.2021.100459

    *Different from the original version, the term “internet-based” was replaced by “digital” in this questionnaire. Moreover “in my work with patients” was added whenever appropriate

    **original: “friend” [↑](#footnote-ref-10)
