# Supplementary material for: Development and evaluation of a web-based toolkit to inform mental health professionals about digital mental health interventions for eating disorders
Source: J Eat Disord. 2026 Jan 9;14:33. doi: 10.1186/s40337-025-01518-1 (PMC12849147; doi:10.1186/s40337-025-01518-1)
Supplement: Supplementary file 3 — Supplementary Material 3 [file 40337_2025_1518_MOESM3_ESM.docx]

**Supplementary Material 3: E-Therapy Attitudes (ETAM) results**

German version:

|  | Item | N | M | SD | Min | Max |
| --- | --- | --- | --- | --- | --- | --- |
| 1 | Digitale Interventionen sind modern bzw. entsprechen unserer heutigen Zeit. | 66 | 3.36 | 0.76 | 1 | 4 |
| 2 | Digitale Interventionen werden konventionelle Psychotherapien zukünftig ersetzen können. | 66 | 0.32 | 0.71 | 0 | 4 |
| 3 | Digitale Interventionen lassen sich besser mit Arbeit und Privatleben vereinbaren als konventionelle Psychotherapien. | 66 | 2.14 | 1.01 | 0 | 4 |
| 4 | Es macht für mich keinen Unterschied, ob eine Psychotherapie digital oder in der Praxis erfolgt. | 66 | 0.3 | 0.55 | 0 | 2 |
| 5 | Digitale Interventionen werden mehr Personen mit psychischen Problemen erreichen. | 66 | 2.89 | 0.96 | 0 | 4 |
| 6 | Krankenkassen sollten die Kosten für digitale Interventionen übernehmen. | 66 | 3.11 | 0.88 | 1 | 4 |
| 7 | Digitale Interventionen sind vergleichbar wirksam wie konventionelle Psychotherapien. | 66 | 0.97 | 0.94 | 0 | 3 |
| 8 | Das Vertrauen zu einer*m Therapeut*in kann digital genauso gut aufgebaut werden wie bei konventionellen Psychotherapien. | 66 | 1.33 | 0.98 | 0 | 4 |
| 9 | Digitale Interventionen sind eine geeignete Alternative zu konventionellen Psychotherapien. | 66 | 1.02 | 1 | 0 | 3 |
| 10 | Bei psychischen Problemen würde ich eine digitale Intervention in Anspruch nehmen. | 66 | 1.94 | 1.18 | 0 | 4 |
| 11 | Ich würde eine digitale Intervention einer konventionellen Psychotherapie vorziehen. | 66 | 0.27 | 0.54 | 0 | 2 |
| 12 | Digitale Interventionen werden mehr Patient*innen erreichen und ihnen helfen können. | 66 | 2.73 | 1.02 | 0 | 4 |
| 13 | Ich mache mir keine besonderen Sorgen um den Datenschutz bei digitalen Angeboten. | 66 | 1.47 | 1.15 | 0 | 4 |
| 14 | Durch die Anonymität bei digitalen Interventionen sinkt die Hemmschwelle, offen und ehrlich über wichtige Probleme zu sprechen. | 66 | 2.33 | 1.01 | 0 | 4 |
| 15 | Durch die Verbreitung von digitalen Interventionen werden sich Menschen früher professionelle Hilfe holen. | 66 | 2.7 | 0.96 | 0 | 4 |
| 16 | Missverständnisse treten bei digitalen Interventionen ähnlich häufig auf wie bei konventionellen Psychotherapien. | 66 | 1.79 | 1.02 | 0 | 4 |
| 17 | Digitale Interventionen eignen sich für die meisten Patient*innen, unabhängig vom persönlichen Hintergrund (Alter, Geschlecht, Bildung, etc.). | 66 | 1.36 | 1.25 | 0 | 4 |

Original version E-therapy attitudes Scale^[[1]](#footnote-1)^; „Internet-based therapies“ have been replaced by „digital interventions“

|  | Item | N | M | SD | Min | Max |
| --- | --- | --- | --- | --- | --- | --- |
| 1 | Digital interventions are modern and in line with our modern times. | 66 | 3.36 | 0.76 | 1 | 4 |
| 2 | Digital interventions will replace conventional face-to-face psychotherapy in the future. | 66 | 0.32 | 0.71 | 0 | 4 |
| 3 | Digital interventions are more compatible with work and private life than conventional face-to-face therapy. | 66 | 2.14 | 1.01 | 0 | 4 |
| 4 | It makes no difference to me whether psychotherapy is conducted through the internet or in a psychotherapy practice in a clinic. | 66 | 0.3 | 0.55 | 0 | 2 |
| 5 | Digital interventions will reach more individuals with mental health problems. | 66 | 2.89 | 0.96 | 0 | 4 |
| 6 | Health insurance companies should cover the costs for digital interventions. | 66 | 3.11 | 0.88 | 1 | 4 |
| 7 | Digital interventions are as effective as conventional face-to-face psychotherapies. | 66 | 0.97 | 0.94 | 0 | 3 |
| 8 | Trust in a therapist can be just as easily built on the internet as in conventional face-to-face psychotherapy. | 66 | 1.33 | 0.98 | 0 | 4 |
| 9 | Digital interventions are an appropriate alternative to conventional face-to-face psychotherapy. | 66 | 1.02 | 1 | 0 | 3 |
| 10 | In case of mental health problems, I would attend a digital intervention. | 66 | 1.94 | 1.18 | 0 | 4 |
| 11 | I would prefer a digital intervention to a conventional face-to-face psychotherapy. | 66 | 0.27 | 0.54 | 0 | 2 |
| 12 | Digital interventions will reach more patients and help them. | 66 | 2.73 | 1.02 | 0 | 4 |
| 13 | I’m not particularly worried about data security in digital interventions. | 66 | 1.47 | 1.15 | 0 | 4 |
| 14 | The anonymity in digital interventions decreases the threshold to speak openly and honestly about important issues. | 66 | 2.33 | 1.01 | 0 | 4 |
| 15 | Through the dissemination of digital interventions, persons will get professional help earlier. | 66 | 2.7 | 0.96 | 0 | 4 |
| 16 | Misunderstandings occur in digital interventions as often as in conventional psychotherapies. | 66 | 1.79 | 1.02 | 0 | 4 |
| 17 | Digital interventions are suitable for most patients, regardless of their personal background (age, sex, education, etc). | 66 | 1.36 | 1.25 | 0 | 4 |

1. Apolinário-Hagen J, Harrer M, Kählke F, Fritsche L, Salewski C, Ebert DD. Public Attitudes Toward Guided Internet-Based Therapies: Web-Based Survey Study. JMIR Ment Health 2018;5(2): e10735. DOI: [10.2196/10735](http://doi.org/10.2196/10735)) [↑](#footnote-ref-1)
